# Boron Nitride Nanotubes: Recent Advances in Their Synthesis, Functionalization, and Applications

**DOI:** 10.3390/molecules21070922

**Published:** 2016-07-15

**Authors:** Chee Huei Lee, Shiva Bhandari, Bishnu Tiwari, Nazmiye Yapici, Dongyan Zhang, Yoke Khin Yap

**Affiliations:** 1 Engineering Product Development, Singapore University of Technology and Design, 8 Somapah Road, Singapore 487372, Singapore; cheehuei_lee@sutd.edu.sg; 2 Department of Physics, Michigan Technological University, 1400 Townsend Drive, Houghton, MI 49931, USA; shivab@mtu.edu (S.B.); bptiwari@mtu.edu (B.T.); nbakca@mtu.edu (N.Y.); dozhang@mtu.edu (D.Z.)

**Keywords:** boron nitride nanotubes, mass production, functionalization, polymer composites, biomedical applications

## Abstract

A comprehensive overview of current research progress on boron nitride nanotubes (BNNTs) is presented in this article. Particularly, recent advancements in controlled synthesis and large-scale production of BNNTs will first be summarized. While recent success in mass production of BNNTs has opened up new opportunities to implement the appealing properties in various applications, concerns about product purity and quality still remain. Secondly, we will summarize the progress in functionalization of BNNTs, which is the necessary step for their applications. Additionally, selected potential applications in structural composites and biomedicine will be highlighted.

## 1. Introduction

BNNTs were first predicted in 1994 [1,2] and experimentally realized in the following year [3]. BNNTs have gained significant attention especially in recent years due to their interesting properties that are not available in other nanomaterials. Although they are structurally analogous to carbon nanotubes (CNTs), BNNTs exhibit completely different physical properties. For examples, BNNTs are electrically insulating with a bandgap of 6 eV [4], while CNTs are either metallic or semiconducting, depending on their chirality and diameter. In addition, BNNTs are chemically and thermally stable up to 800 °C in air. BNNTs offer excellent thermal conductivity [5], a high Young’s modulus (up to 1.3 TPa) [6] and superhydropbobicity [7,8]. More recently, the Young’s modulus and shear modulus were determined to be 1.8 ± 0.3 TPa and 7 ± 1 GPa [9]. BNNTs are also predicted to be piezoelectric [10,11,12] and may be useful for spintronic devices [13]. However, there is a consensus that BNNTs are very difficult to synthesize. For the past two decades, the research progress on BNNTs has been hindered by the limited quantity and quality of BNNTs samples available for widespread investigation of their properties and industrial applications [14,15]. In this article, progress on controlled synthesis and large scale production of BNNTs will be presented. Functionalization and potential applications of BNNTs in composites and bio-medicine will then be discussed. It is noted that electrically insulating BNNTs were recently being used for applications in digital switches when functionalized with metallic quantum dots [16,17] and graphene [18]. However, electronic device application will not be reviewed here at this early stage of the research.

## 2. Synthesis of BNNTs

The synthesis of BNNTs by various techniques has been demonstrated. Pioneering and early works on BNNTs were mainly inspired by the techniques for CNT synthesis, including arc-discharge [3,19], laser heating and vaporization [20,21,22,23], the BN substitution method from CNT templates [24,25], chemical vapor deposition (CVD) using borazine [26,27], induction heating boron oxide CVD (BOCVD) [4,28,29,30] and high-temperature ball milling [31,32]. Comprehensive reviews on synthesis processes can be found elsewhere [14,15,33,34]. In this section, controlled synthesis and mass production of BNNTs will be highlighted and discussed.

### 2.1. Thermal Annealing and Chemical Vapor Deposition

Among different chemistry pathways using the CVD approach, a novel and effective process was invented by Bando’s group in Japan. In 2002, Tang et al. [28] found an effective chemical pathway to produce BN precursor for the synthesis of BNNTs. Together with boron (B), metal oxides (such as MgO, FeO, Li_2_O) have been found to be effective precursors to produce reactive boron oxide (B_x_O_y_) vapour to react in ammonia (NH_3_) environment. An induction heating chamber was used to initiate the chemical reaction at 1300 °C for the synthesis of BNNTs. This BOCVD technique has been further improved by Zhi et al. [29] and is capable in producing BNNT with reasonably low impurities [35]. This process was very successful for producing BNNTs in gram amounts in a laboratory environment [15]. Due to the availability of these BOCVD BNNTs, the research group has further initiated other related works, such as purification, dispersion and functionalization, doping, polymeric composites and so on. Some of the results will be discussed in the following sections. However, commercialization and real application of BNNTs in industry is hindered by its relatively lower production rate and the need for customized chamber design.

**Figure 1:**
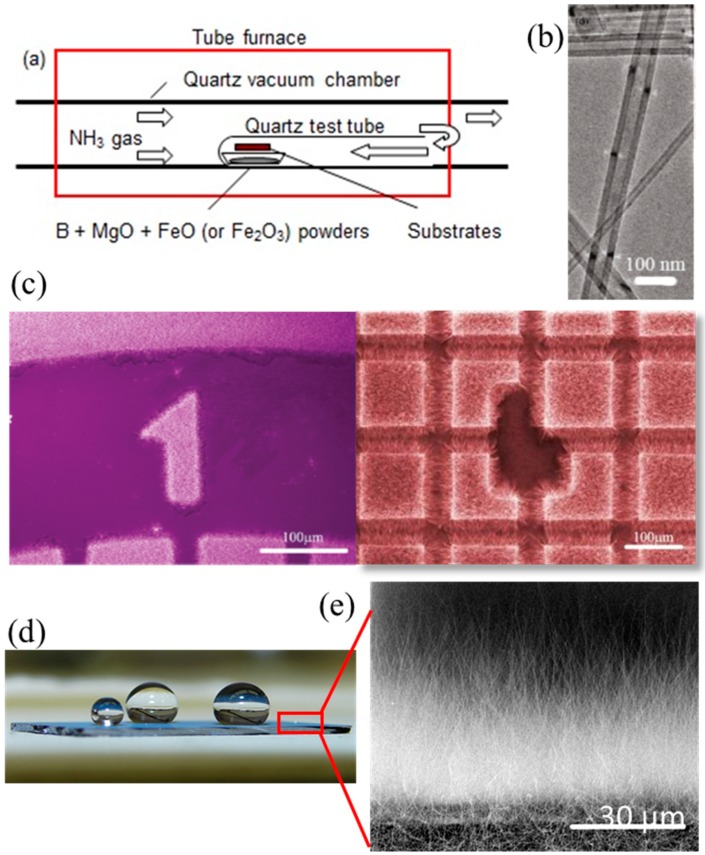
(**a**) Experimental setup for the growth of BNNTs in a horizontal tube furnace. Adapted with permission from [30]. Copyright 2008 IOP Publishing Ltd.; (**b**) TEM image showing tubular structure of BNNT with amorphous free side walls; (**c**) Well-defined patterned growth of BNNTs on a substrate. Adapted with permission from [4]. Copyright 2010 American Chemical Society; (**d**) Photograph of water droplet on the surface of BNNT film produced by CCVD showing superhydrophobicity; (**e**) Zoom-in cross sectional view of BNNTs on Silicon substrate showing that these BNNTs are grown vertically aligned on the substrate surface. Adapted with permission from [8]. Copyright 2012 American Chemical Society.

Using a similar chemistry, Lee et al. demonstrated the production of high-purity BNNTs at 1200 °C in a conventional resistive tube furnace [30]. As illustrated in Figure 1a, the key feature of this catalytic CVD (CCVD) technique is the use of a closed end quartz test tube to trap and confine the growth vapours for the formation of BNNTs. This technique is called the growth vapour trapping (GVT) approach. The GVT growth of BNNTs can be controlled by the vapour-liquid-solid (VLS) process by the use of catalytic nanoparticles (MgO, Fe, Ni) coated on Si substrates. This controlled approach is significantly different from the original BOCVD, where BNNTs are merely formed by spontaneous nucleation/condensation. Based on the VLS process, patterned growth of BNNTs on a Si substrate by CVD was reported for the first time [4]. At optimized growth condition, this CCVD/GVT approach has led to the growth of high quality and high purity BNNTs at desired locations predefined by catalyst coatings (see Figure 1b,c). Patterned growth not only explains the role of catalysts in the formation of BNNTs, but it also opens an opportunity to directly fabricate BNNT devices on substrates at controllable locations. In addition, the GVT method can be easily reproducible in many other research labs, as the setup is relatively simple [36]. These as-grown BNNTs by this CCVD/GVT technique are vertically-aligned (Figure 1e), with a band gap of 6 eV without any sub-band features. Interestingly, these BNNT films exhibit super-hydrophobicity due to its nanoscopic surface roughness (Figure 1d) [7] and reduced surface energy due to adsorbate formation [8].

On the other hand, the high temperature ball milling process was another synthesis approach for BNNTs as initiated by Chen et al. in 1999 [31]. This process has been further developed and refined using boron ink [32,37,38,39]. In brief, boron powder was ball milled under N_2_/NH_3_ atmosphere for a long duration (up to 150 h). Later, they were mixed with ferric nitrate (Fe(NO_3_)_3_) and cobalt nitrate (Co(NO_3_)_2_) in ethanol to make B ink. After that, this solution was further treated with N_2_/NH_3_ between 1000 °C and 1300 °C for several hours to convert it into BNNTs. B ink could also be applied on various surfaces to deposit BNNTs [38].

The typical diameter of BNNTs produced by the above mentioned CVD and thermal annealing methods is of 50–80 nm, and the length is of 100–200 μm. The BOCVD and CCVD/GVT approaches produce multi-wall nanotubes with very high quality tubular structure. On the other hand, many of the as-grown BNNTs by ball-milling have a bamboo-like structure, potentially due to the contamination of metal particles from the steel milling balls. The intrinsic properties of such bamboo-like BNNTs may be different from those with tubular structures. The boron ink approach seems to overcome the bamboo-like issue.

### 2.2. Laser Vaporization with Pressurized Vapor/Condenser Method

Inspired by CNTs growth, the laser ablation or laser evaporation technique has been employed for BNNTs growth since 1996. Pioneering work was performed by Golberg et al. [40], and followed by other works [20,22,23,41]. In 2005, Yap et al. demonstrated the first success in patterned growth of BNNTs on substrates by iron catalysts using pulsed-laser deposition of a BN target [42]. This catalytic PLD approach has led to the formation of fine BNNTs (~10–20 nm in diameter) at a significantly low substrate temperature of 600 °C. In 2007, Arenal et al. reported the root-based growth mechanism of single wall BNNTs by laser heating at 1 atm under N_2_ [20]. This approach successfully produced single walled (or few walled) BNNTs. However, it suffered from significantly low yield and high concentrations of B impurity.

**Figure 2:**
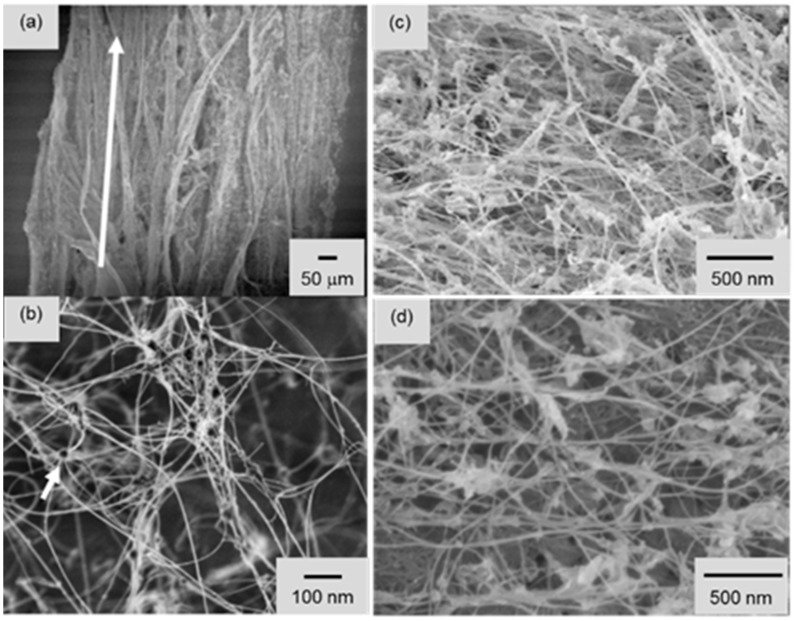
SEM images of (**a**) BNNT fibrils and (**b**) the entangled BNNT network produced by the pressurized vapour/condenser method (PVC). The white arrow in (**a**) indicates the growth direction, parallel to the BNNT fibrils. The short arrow in (**b**) marks a round, solidified boron droplet in the network. Adapted with permission from [43]. Copyright 2009 IOP Publishing Ltd.; (**c**) Entangled BNNTs within the BNNT fibrils grown from hydrogen catalysed inductively coupled plasma. Adapted with permission from [46]. Copyright Published 2014 by American Chemical Society; (**d**) Image of entangled BNNT fibril produced by the EPIC system. Adapted with permission from [47]. Copyright 2014 American Chemical Society

**Figure 3:**
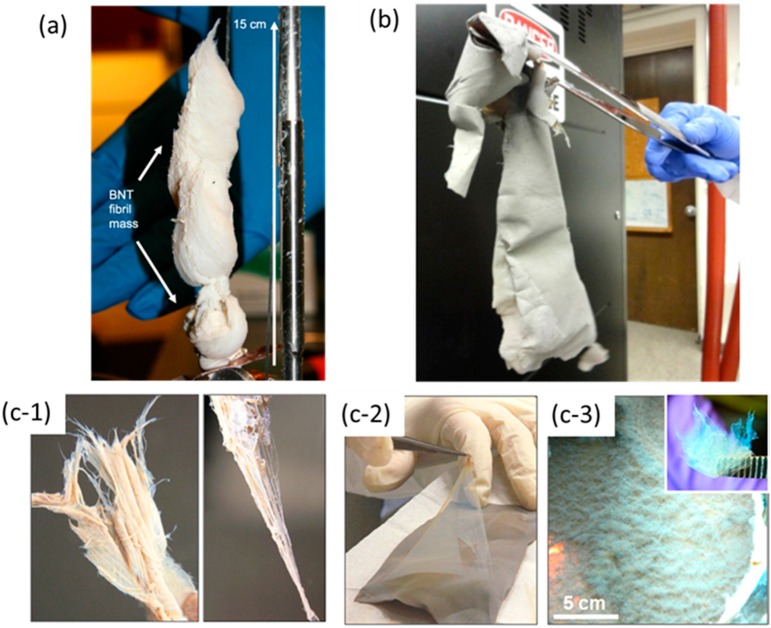
Macroscopic photographs of BNNTs produced by different techniques: (**a**) modified laser vaporization PVC/HTP method; (**b**) EPIC method; (**c**) Inductive thermal plasma. (**a**) The results of a 200 mg BNNT by PVC production. The unprocessed material has the appearance of cotton balls, though the texture is somewhat softer and the material finer-grained. Adapted with permission from [43]. Copyright IOP Publishing Ltd.; (**b**) Production via the EPIC method. BNNT felt-like sheet peeled intact from the walls of the reaction chamber. Adapted with permission from [47]. Copyright 2014 American Chemical Society; (**c**) BNNT materials grown by an induction plasma process. The as-grown BNNT materials exhibit three distinct morphologies: (**c-1**) entangled fibril materials consist of many fibrils formed naturally, and BNNT yarns can be drawn directly from them; (**c-2**) Cloth-like sheets have a multi-layered structure, and thin diaphanous membranes can be easily peeled off; (**c-3**) Fluffy cotton-like deposits have a low density and cover the entire wall of the filtration chamber. Inset is a close-up image. Adapted with permission from [46]. Copyright Published 2014 by American Chemical Society.

A significant improvement in BNNTs synthesis was reported in 2009 using a modified laser evaporation technique [43]. This approach utilized the pressurized vapor/condenser method (PVC) and later was referred as the high temperature/high pressure (HTP) method. In this work, a high-powered laser of 1 kW free-electron laser (wavelength of 1.6 μm) or a kW-class CO_2_ laser (wavelength of 10.6 μm) was used to vaporize a boron (or BN) target inside a chamber filled with high pressure nitrogen (pressure of 2–20 bars). An upward stream of hot boron vapor (~4000 °C) can be produced and condensed into liquid boron droplets which serve as nucleation sites. This approach is quite similar to those previously demonstrated except for much higher laser powers and N_2_ pressure. The B droplets generated by the laser further translate upwards due to the elevated pressure (N_2_) and quickly form BNNTs. A cooled metal wire traversed through the boron plume in the chamber acts as a condenser to promote the growth of BNNTs fibrils. The condenser can be made from BN, B, stainless steel, copper niobium (Cu-Nb) and tungsten (W) in various shapes of wires, sheets, ribbons and rods. The throughput or production rate is typically from 20 to 120 mg/h. As grown fibrils are the tube bundles and entangled network of BNNTs (Figure 2a,b). The majority of BNNTs have 2 to 5 walls. Figure 3a shows the cotton-balls like BNNTs by the PVC/HTP method. As shown, staining of boron particles, and non-BNNT boron nitride (BN_x_) by-products are visible and significant.

The PVC/HTP method was recently modified with a new high pressure (1000 psi or 68 atm) chamber using a 2.5 kW diffusion cooled CO_2_ slab laser [44]. Theoretical simulation [45] and in-situ diagnostics were applied to develop a detailed understanding of the chemistry and the growth mechanism of BNNTs [44].

### 2.3. Large Scale Synthesis by Inductive Thermal Plasma Method

In 2014, a customized inductively coupled thermal plasma reactor (with commercial available plasma torch) was employed for large-scale synthesis of small-diameter (~5 nm) BNNTs at an impressive rate of 20 g/h. Solid hexagonal boron nitride (h-BN) powder was fed along with N_2_ and H_2_ gases in a high temperature induction plasma (>8000 K) at atmospheric pressure [46].

As the high temperature plasma decomposed all the precursor materials into their constituent elements (B, N and H), nano-sized boron droplets were condensed in the cooler downstream of the reactor due to the large temperature gradient (10^5^ K/s) and further acted as nucleation site to form BNNTs. It was claimed that hydrogen gas was necessary as the catalyst by forming an intermediate H-B-N species so that it hinders the recombination of N radicals generated from N_2_ feedstock or from dissociation of h-BN. These intermediate species can easily result into h-BN like phase to nucleate BNNTs from the boron droplets. Similar to PVC/HTP method, the macroscopic morphologies of the product could appear in the entangled network of BNNT fibrils, cloth like sheet and fluffy cotton deposit. Figure 2c shows an SEM image of the morphology of entangled BNNT fibrils. However, as seen in SEM images, the product contained non-tubular structured impurities, such as h-BN fragments, amorphous boron, amorphous boron nitride, BN_x_ by-products and possibly other ternary B-N-H compounds. These impurities are entangled with BNNTs into various fibril structures as shown in Figure 3c.

A similar inductively coupled plasma technique for the synthesis of BNNTs was independently reported in 2014 [47]. Interestingly, hydrogen is not necessary for this approach. A customized extended pressure inductively coupled plasma (EPIC) system was built to produce plasma with power between 40 to 50 kW. A N_2_ plasma plume was generated by flowing N_2_ gas at 50 L per minute. Boron feedstock (amorphous boron or h-BN powder) were injected at a rate of 100 mg/min to 1700 mg/min using N_2_ as the carrier gas (2–5 L/min) into the plasma plume at pressures varying from 14.7 to 75 psi absolute. At high N_2_ pressure, molten boron droplets formed within plasma plume react with N_2_ to form BNNTs, yielding a record growth rate of 35 g per hour. The product material contained mostly small diameter double-walled BNNTs with a diameter of ~4 nm. It should be noted that quenching rates in the reaction chamber is an important parameter in the synthesis process. Figure 2d shows the morphology of these entangled BNNTs with high contrast spots attributed as unreacted solidified boron droplets or BN_x_/non-BNNT impurities. Figure 3b shows BNNT fibrils produced by EPIC with the synthesis conditions: amorphous boron at 246 mg/min; carrier gas N_2_ at 2.5 L/min; plasma gas N_2_ at 50 L/min; 40 kW plasma at 30 psia.

### 2.4. Summary Remarks on Controlled Synthesis and Mass Production of BNNTs

Recent developments in controlled synthesis and mass production of BNNTs have been significant. Here, several remarks are summarized on the failure of mass production [15] prior to 2009 and the key aspects of the current mass production:

1. In contrast to the CVD method, all the plasma-based techniques (both laser plasma and inductive plasma) utilize very high synthesis temperatures (4000 K to 8000 K). These approaches led to the vaporization of B or BN solids into molten B droplets that induce the formation of BNNTs.
2. High pressure environment (>1 atm) of nitrogen effectively facilitates and increases the formation of BNNTs from B droplets, due to higher collision rate of N_2_ or N radical with B droplets.
3. A large temperature gradient (i.e., the high cooling rate) by drastic velocity profile or spatial profile (e.g., a solid condenser or quench rate) seems to enhance the formation small B droplets and hence further promotes long and small-diameter BNNTs formation.
4. Apparently, the drawbacks of these plasma-based techniques are as follows:
  (a) Excessive high synthesis temperatures have led to serious contamination and clustering of BNNTs. This will make purification and dispersion of the fibril-like BNNTs challenging.
  (b) The plasma-based approaches required a customized chamber to generate high synthesis temperature, high pressure and rapid cooling conditions for mass production.

Obviously, all these plasma techniques failed in producing high-purity BNNTs. Purification by oxidation will lead to structural damage and cutting of BNNTs. In contrast, the CCVD/GVT approach (Figure 1) is capable of producing high-quality and high-purity BNNTs even though the production rate is still low. Nevertheless, mass production and commercialization of BNNTs are now in place. Therefore, researchers and engineers can obtain samples in gram quantity for their research activities and product development [48,49]. The current commercial BNNTs are produced either by the PVC/HTP method or inductively coupled thermal plasma. The current price of few-walled BNNTs is set within USD 100 per 100 mg range. It is expected that more vibrant research on BNNTs will be stimulated due to the availability of the material. Industrial and real world applications of BNNTs will soon become feasible.

### 2.5. Purification, Dispersion and Functionalization of BNNTs

Although mass production of BNNTs has become feasible, impurity is still a critical issue. Bulk BNNTs with high purity are snow-white in appearance [15], while brownish or grayish samples (as shown in Figure 3) provide an indication of impurity within the BNNT fibrils. Purification is necessary for many applications, such as those in the areas of composites, and bio-medicine.

Several approaches have been explored to purify as-grown BNNTs contaminated with metallic catalysts, unreacted precursors and other non-BNNT boron nitride (BN_x_) by-products, etc. Overall, there are three common categories of purification processes: (1) acid treatment [50], (2) thermal oxidation/annealing in air [46], in addition to (3) surfactant or polymer wrapping separation via functionalization [51]. These purification processes are usually introduced after synthesis. Even though BNNTs are chemically inert and resistive to oxidation up to ~900 °C, one should be mindful that rigorous purification process, such as sonication and strong acid treatment will lead to damaging and cutting of the BNNT structure and hence altering their intrinsic properties [52,53]. As compared to metallic particles, BN_x_ impurities would be more difficult to remove since they are also chemically stable and oxidation resistant. 

The dispersion of nanomaterials in liquids is also crucial for many applications. Because of their small sizes, nanomaterials tend to interact by van der Waals forces. Hence they have a tendency to aggregate in liquids. The similar scenario happens to BNNTs, especially long BNNTs tend to entangle with each other. It has become indispensable to develop techniques to disperse and stabilize BNNTs in aqueous and non-aqueous media for applications in composite, and biomedical areas. The dispersion of pure BNNTs (without chemical functionalization) with a series of solvents was recently studied based on the thermodynamic Hansen parameters [54]. It was found that dimethylacetamide (DMAc) can produce the most stable and uniform dispersion of BNNTs, followed by *N*,*N*-dimethyl-formamide (DMF), acetone, and *N*-methyl-2-pyrrolidone (NMP). This study further proposed and examined a new idea of co-solvent systems to improve the dispersion of BNNTs [54]. This approach enables the use of various organic solvents and polymers for the fabrication of BNNT composites, thin films, coatings, and paints.

In addition to polar organic solvents, functionalization has become a common practice to stabilize BNNTs in aqueous media. Two main approaches, namely covalent and non-covalent functionalization have been established by many research groups. Indeed, other modification approaches have also been developed over the years, including doping with exotic elements and BCN nanotubes [55,56,57,58], filling of metals or semiconductors into the tubular structure [59,60], as well as nanoparticle decoration on the surfaces of BNNTs [16,61,62,63]. In the following sections, non-covalent and covalent functionalization will be discussed as they are relatively promising for large scaled applications.

## 3. Non-Covalent Functionalization

Weak interactions such as van der Waals force, electrostatic interaction and π-stacking (π-π) interaction can be utilized to attach polymers or functional molecules on the surfaces of BNNTs. These non-covalent functionalization methods are advantageous than covalent functionalization because of their simplicity. Non-covalent functionalization can also likely preserve the intrinsic properties of BNNTs, although this may not be always true, depending on the type of interaction involved. In this context, π-stacking interaction between BNNTs and conjugated polymers [64] or aromatic molecules [65] is considered as the effective approach for BNNT dispersion in solvents. This is because of the presence of π-electrons on the hexagonal BN network of BNNTs with the π-electrons localized at N atoms (polarized or ionic B-N bonds). A series of reports on non-covalent functionalization was reported by Zhi et al. [51,66,67,68,69,70], with the availability of BNNTs produced by BOCVD. Readers can refer to a series of review articles for comprehensive details [15,34,71,72]. 

BNNTs in aqueous media form bundles and inhomogeneous aggregates [70]. This affects the cellular uptake processes (internalization/endocytosis) and reliability of biocompatibility investigations [73]. To address this problem, several approaches have been demonstrated to functionalize BNNTs in water. For example, BNNTs were functionalized by utilizing the electrostatic interaction between boron on the BNNTs and amino groups of poly-(ethyleneglycol) (NH_2_-PEG1500) [74]. Furthermore, Zhi et al. showed protein-BNNT interaction in a controllable manner [75]. In their study, while ferritins can be localized in the hollow core of BNNTs in absence of any additional chemical, it can also be immobilized onto BNNTs via 1-pyrenebutyric acid N-hydroxysuccinimide ester (PAHE). In the latter case, BNNTs interact with pyrene- via π-π stacking and the NHS- (N-hydroxysuccinimide ester) on PAHE reacts with amino groups of ferritin.

Ciofani et al. use BNNTs for biomedical applications by investigating various functionalization approaches in water. BNNTs were successfully dispersed and functionalized in glycol-chitosan (GC) [76,77], poly-l-lysine (PLL) [78,79] and polyethyleneimine (PEI) [80]. Cytocompatibility of those functionalized BNNTs were further investigated and results showed dependence on the polymer used [81,82]. Another GC-BNNTs complex was also studied by Soares et al. by a slight modification of the previous approach. They were able to functionalize BNNTs with GC by mixing in ethanol, stirring at 70 °C, purified and dispersed in water by mild ultrasonication process [83]. Chen et al. have also utilized the amphipathic dendritic structures to form a stable suspension of BNNTs in water. The synthetic carbohydrate ligands present at the chain end of the dendrimers can be specifically bound to receptors. Second generation dendrimer (G-2) with R-mannose moieties (G-2 Man) was selected to functionalize BNNTs [84]. Gao et al. exploited the π-π interaction of a peptide named B3 (HWSAWWIRSNQS) for forming B3/BNNTs stable complexes [85]. B3/BNNTs suspensions were obtained by simple sonication procedure. Formation of B3 coating on BNNTs was confirmed by AFM images as the diameter of BNNTs after functionalization increased to ~80 nm from 20–50 nm. FTIR and UV-Vis spectroscopy further confirmed the formation of B3/BNNTs complexes. It has been shown that the electrical structure of BNNTs was altered by the coating.

**Figure 4:**
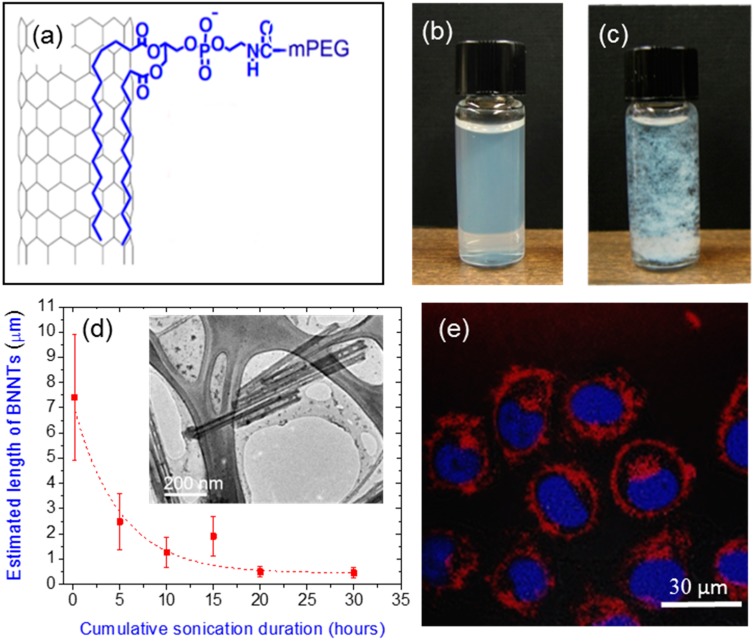
(**a**) Schematic of BNNTs functionalized with m-PEG-DSPE molecules in water. (**b**) Appearance of well-dispersed BNNTs functionalized with mPEG-DSPE molecules in water. These suspension is very stable versus unfunctionalized BNNTs suspended in ethanol (**c**); (**d**) The lengths of the functionalized BNNTs as a function of sonication/cutting duration. TEM images of cut BNNTs are shown in the inset. Adapted with permission from [52]. Copyright 2010 American Chemical Society; (**e**) Overlay of fluorescence images of HeLa cells (nuclei stained in blue) incubated with functionalized BNNTs (in red).

Lee et al. exploited the hydrophilic and hydrophobic properties of a biocompatible polymer methoxy-poly (ethylene glycol)-1,2-distearoyl-sn-glycero-3-phosphoethanolamine-*N* conjugates (mPEG-DSPE; MW 5000 Da) for the functionalization of BNNTs [52]. The mPEG-DSPE molecules are a type of PEGylated phospholipids and can be attached on BNNTs as schematically shown in Figure 4a. BNNTs functionalized with mPEG-DSPE are stable in water for a few months, as shown in Figure 4b versus the un-functionalized BNNTs suspended in ethanol as shown in Figure 4c. It was further shown for the first time that the length of the functionalized BNNTs can be cut and shortened to less than 500 nm via rigorous sonication (Figure 4d). Based on controlled experiments, the authors suggested that the DSPE tail of mPEG-DSPE can adsorb and wrap around the BNNTs walls by the van der Waals forces, charge transfer or hydrophobic forces. On the other hand, the hydrophilic PEG groups interact with water molecules in forming a highly stable suspension of mPEG-DSPE/BNNTs. More recently, the same group shows that cut BNNTs are biologically compatible in HeLa cells. A confocal microscopy overlay image of these BNNTs in HeLa cells is presented in Figure 4e.

## 4. Covalent Functionalization

Covalent functionalization on BNNTs is relatively challenging due to the chemical inertness of BNNTs. Since all B and N atoms within the sp^2^ hexagonal network of BNNTs are covalently bonded, defects or dangling bonds must be created on BNNTs prior to covalent functionalization with other molecules. Therefore, covalent functionalization can potentially alter the intrinsic properties of BNNTs. Zhi et al. introduced reaction between -COCl group of stearoyl chloride and the amino groups generated on the defect sites of BNNTs. The modification was done by refluxing the mixture for 120 h at 100 °C. Modified BNNTs were dispersible in solvents such as chloroform, *N*,*N*-dimethyl-acetamide, tetrahydrofuran, *N*,*N*-dimethylformamide, acetone, toluene and ethanol [86]. Various other methods have been developed for the covalent modifications of BNNTs such as heating BNNTs with amine terminated poly(ethylene glycol) which formed ionic bonds with boron sites on BNNTs [74], cycling treatment of BNNTs with dimethyl sulfoxide (DMSO) to peel B-N bond [87] and by fluorination during the BNNTs synthesis [88]. Similarly, Ciofani et al. have also demonstrated the covalent approach to functionalize BNNTs by grafting amino functional group on the surface of BNNTs [81]. In their approach, BNNTs were first dispersed and sonicated in HNO_3_ solution to introduce -OH groups at the surface of BNNTs via a strong oxidation process. The oxidized BNNTs were further sonicated in 3-aminopropyltriethoxysilane (APTES) which reacts with the hydroxyl group on the surface of BNNTs [89], thus resulting free amino groups on the nanotubes side walls allowing further conjugation to form BNNT complexes. As claimed, these covalent modifications of BNNTs may open ways for prospective applications of BNNTs in drug delivery such as in cell targeting and in general nanomedicine research. 

Recently, reduction chemistry was proposed as an intermediate step for covalent functionalization of BNNTs [90]. As assisted by density functional theory (DFT), covalent alkylation of BNNTs was demonstrated using 1-bromohexane by first performing the reduction chemistry. The reduction of BNNTs was conducted using sodium naphthalide salt in tetrahydrofuran (THF) solution. The thermogravimetric (TG) analysis and Fourier Transform Infrared (FTIR) measurements showed a significant increase in reactivity of reduced BNNTs toward covalent alkylation compared to pristine samples.

## 5. Potential Applications

### 5.1. Nano-Fillers for Composites

BNNTs are highly favorable in nanocomposites for mechanical applications due to their excellent chemical inertness, high thermal conductivity, strong mechanical properties and electrically insulating nature. BNNT composites have the potential as nano-filler for structural reinforcements of composite materials. In early studies, for example, a ~1 wt % of soluble BNNTs (with PmPV surfactant) was used in polystyrene (PS) composite exhibited 21% increase in the elastic modulus [91]. In addition, the composite displayed perfect transparency. Up to 10 wt % of BNNTs can be incorporated into polymethyl methacrylate (PMMA) polymeric matrix to improve the thermal conductivity by ~3-fold [15].

Ceramic-BNNTs composite were also reported, but the publications in this area are quite limited. A barium calcium aluminosilicate (BCAS) glass composite containing ~4 wt % BNNTs was fabricated by hot pressing. It was found that the flexure strength and fracture toughness of the composite was increased by 90% and 35%, respectively compared to those of the intrinsic glass. In addition, Al_2_O_3_-BNNTs and Si_3_N_4_ ceramic-BNNTs composites were also reported [92]. With a small addition (0.5 wt %) of BNNTs, the Vickers hardness of Al_2_O_3_ can be increased from 17.3 to 19.1 GPa. However, with higher fraction of BNNTs, the value dropped to 14.5 GPa.

As inspired by CNTs-metal composites, BNNTs-aluminum matrices were investigated with the aim of making lightweight and strong Al matrices. This was performed using a powder metallurgy technique-a high pressure torsion processing technique (HPT) at room temperature under 5 GPa pressurization [93]. For 5 wt % of BNNT incorporation (annealed at 450 °C), it was found that the tensile strength of the Al matrices is ~420 MPa at room temperature, more than two times higher than that of pure Al samples fabricated under the identical conditions. It should be noted that the result can be process-dependent. In another study by using spark plasma sintering (SPS), the hardness of the composites was not changed with the increase of BNNTs content [94].

There has been a great deal of research interest in polymeric BNNT composites. For example, a filtering-absorbing process was reported to allow the incorporation of BNNT (up to 18–37 wt % BNNTs) into polymer matrices. In this study, four different types of polymer were used, including polyvinyl butyral (PVB), polystyrene (PS), polymethyl methacrylate (PMMA) and polyethylene vinyl alcohol (PEVA). It was shown that thermal conductivity of PMMA can be improved over 21-times [95]. More recently, it was reported that one can incorporate up to 75 wt % of BNNTs into polymers [44]. Further investigation is needed to understand the mechanical performance of these composites.

There is a growing interest in using BNNT composites for shielding applications. For example, BNNT composites may be useful as ultra-violet (UV) filters without affecting the transmission of visible light. This is due to the fact that BNNT strongly absorb UV radiation due to their wide band gap property. In addition, BNNTs or BN materials with isotope boron-10 (^10^B) have attracted significant research interest in neutron radiation shielding, especially in space and aerospace industries and nuclear engineering [96]. Besides helium-3 (^3^He), ^10^B has one of the largest neutron absorption cross section (3835 barns) among all elements [97]. It is worth mentioning that secondary neutrons, which have been previously ignored, can be produced inside a space structure when the space radiation (such as galactic cosmic radiation, solar energetic particles, etc.) interacts with the walls of the structures. BNNTs have potential to be a multifunctional material, not only for their light weight yet strong mechanical structure, but also for space radiation and neutron radiation shielding [44].

#### Remarks on Composite Application of BNNTs

As BNNTs can now be produced in larger quantity, industrial applications of BNNT composites become more possible. The incorporation of BNNTs is capable of introducing multiple property enhancements simultaneously, including the mechanical strength, neutron radiation shielding, and thermal conductivity. Overall, better composite properties can be obtained by the incorporation of BNNTs due to their higher elastic modulus, and higher heat conductivity. However, higher fraction of BNNTs in the polymeric composites does not guarantee higher performance of the composites, likely due to the agglomeration of the nanotubes. Apparently, in the composite fabrication process, the quality and the purity of BNNTs can affect the performance of the composites. There is a plenty of room for improvement in the current mass production techniques by overcoming the quality and purity issues of BNNT fibrils. In contrast, current CCVD approach for controlled synthesis of BNNTs produce high-quality BNNTs in high purity. However, large-scale synthesis by CCVD is still in its infancy regarding its ability to meet the needs of composite materials production. 

### 5.2. Biomedical Applications

Over the past two decades, there has been great interest and enthusiasm for nanomaterials in biomedical applications. This is due to their small size, high surface to volume ratio, surface functionality, in combination with other physical, chemical, and biological properties of the nanomaterials [98]. For the same reasons, BNNTs have attracted significant research interest for biomedical applications [99]. For example, BNNTs can be incorporated into polylactide-polycaprolactone (PLC) copolymer, a biodegradable polymer to improve the mechanical properties of the polymer for orthopedic implant/scaffold application [100]. Incorporation of 5 wt % BNNTs in PLC was found to increase the elastic modulus of PLC by 1370% increase and the tensile strength of PLC by 109%. An increase in osteoblast cell viability was also observed with the BNNT-PLC composite, compared to that of pure PLC [100]. The incorporation of BNNTs also resulted in the increase of the *Runx2* gene expression level, the main regulator of osteoblast differentiation. Hydroxyapatite (HA, Ca_10_(PO_4_)_6_(OH)_2_), a clinically accepted orthopaedic implant material with a crystal structure similar to apatite in human skeleton, exhibits poor fracture toughness and wear resistance for application in load bearing orthopaedic implants. In order to overcome this limitation, BNNTs were added into HA composite for mechanical reinforcement [101]. The hardness, fracture toughness and wear resistance of the composite were found to improve, without adverse effect on the osteoblasts cell proliferation and viability. In another recent work, BNNTs were demonstrated to reinforce β-tricalcium phosphate (β-TCP) scaffolds that are manufactured using laser sintering. In this study, BNNTs were found to promote the osteogenic differentiation of umbilical cord mesenchymal stem cells (UC-MSCs), indicating good osteoinductive properties for potential use in bone scaffolds and repair [102].

**Figure 5:**
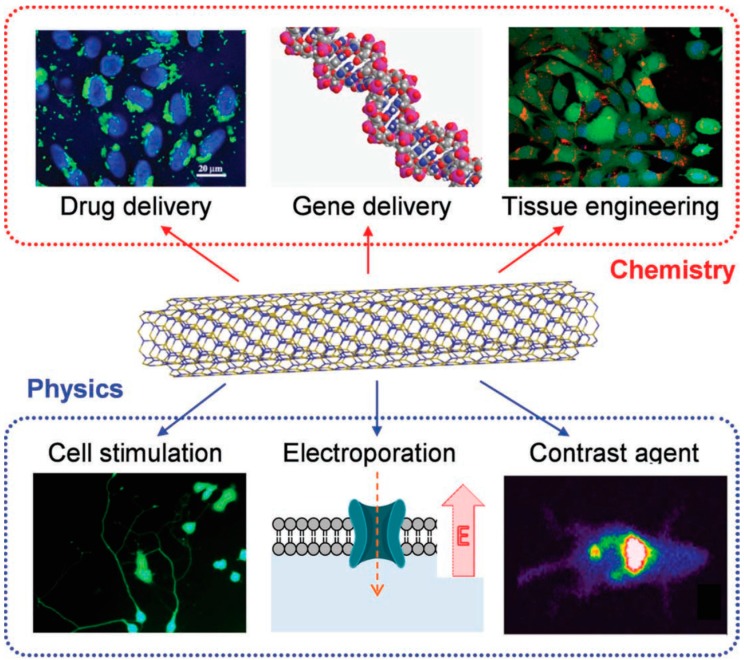
Schematic representation of potential applications of BNNTs in biomedicine. Adapted with permission from [81]. Copyright 2013 Wiley-VCH Verlag GmbH & Co. KGaA, Weinheim.

For biomedical applications, cytocompatibility of novel nanomaterials by in vitro studies is always the first step to be accomplished. Eventually, in vivo testing is mandatory in order to proceed before any realistic biomedical application. Indeed, biocompatibility studies of BNNTs usually relate to functionalization and dispersion of BNNTs in aqueous solution. Ciofani et al. and Chen et al. initiated the first biocompatibility tests on BNNTs [80,84]. Several review articles on the cytotoxicity, biocompatibility and biomedical application studies of BNNTs can also be found in the literature [34,71,81,99,103,104]. Figure 5 illustrates the current trend of potential BNNTs applications in nanomedicine.

Most of the biocompatibility evaluations were performed by introducing functionalized BNNTs into the cell culture media in vitro. Recently, in vivo toxicological studies with different biological systems have been initiated and the results have been promising [83,105,106]. In brief, multiple injection (once per day for three days) of 5 mg/kg of GC-BNNTs were very well tolerated by the investigated rabbits, without evidence of adverse effects, including no blood, liver or kidney impairment [105]. For the first time, the pharmacokinetic analysis of BNNTs in organism was also performed and the blood half-life of GC-BNNTs was calculated to be ~1.5 h [105]. The data are encouraging, and suggest the suitability and biocompatibility of BNNTs for pharmaceutical applications.

Besides in vitro and in vivo studies, cell culture was performed on BNNT films coated on steel substrates by the B ink method. These BNNT films were first transformed from hydrophobic to hydrophilic after plasma treatments. The cell culture tests were performed by using human mammary fibroblasts and transformed cell line (TXP RFP3). Results showed that pristine BNNT films could support the cell growth. On the other hand, plasma treatments can enhance the cell adhesion and proliferation, attributed to the surface roughness of the 3D nanostructured BNNT films. It was claimed that BNNT films can be useful as scaffold for tissue engineering applications [107].

Boron neutron capture therapy (BNCT) is a selective radiotherapy where ^10^B atoms are used to absorb low-energy thermal neutrons, which will then spontaneously decays to produce ^4^He (α particle) and ^7^Li. In principle, it has the potential for effective treatment for many forms of cancers [108,109]. Because of these high linear energy transfer, the resultant α particles have limited path lengths in tissues and cells (5–9 μm). Therefore, the destructive effect of α particles is limited to boron containing cells. In order for BNCT to be successful, a sufficient amount of ^10^B must be selectively delivered to the tumor cells (~20 μg/g or ~10^9^ atoms/cell), and enough thermal neutrons must be absorbed by them to sustain a lethal ^10^B (n,α) ^7^Li capture reaction [110]. BNCT has progressed relatively slowly since 1950s [111]. The research community has been actively looking for effective boron delivery agents, a difficult task of the highest priority [110]. As of now, sodium borocaptate (BSH) and boronophenylalanine (BPA) compounds are the only common boron delivery agents, although other boron-contained compounds are also being studied [110]. Not much attention is paid to BNNTs so far. The advantages of using BNNTs are their simple chemical composition (B and N) and chemical inertness. More importantly, it can be functionalized to recognize specific tumors. Very recently, the BNNT biomedical research community has initiated studies and produces promising results, by investigating functionalized BNNTs as boron atom carriers to targeted cells in vitro [112,113,114]. Surely, for real BNCT applications, more work needs to be done. To enable the application of BNNTs in BNCT, ^10^B must be used for the synthesis of BNNTs, which is experimentally very feasible. 

#### Remarks on Cytotoxicity of BNNTs in Biomedical Applications

Much like the cases of other nanomaterials, the cytotoxicity of BNNTs could depend on their purity, concentration, functionalization, size, and structure (presence of defects), as well as the cell type that is investigated. We notice that BNNT biomedical research reported so far are based on the use of BNNTs with inconsistent sample quality (such as bamboo-like structures, large BNNT diameter >50 nm, amorphous-like rough tube surface) and purity (samples with high fraction of impurities and byproducts, etc.). Therefore, controversial results (sometimes contradicting with each other) were reported regarding cytotoxicity of BNNTs in biological system [81,82]. The lack of high quality BNNTs in the past is the leading cause of inconsistency in BNNT biomedical research.

Apparently, there is still no systematic toxicity studies on the effect of sample quality and purity, as well as a comparison between single-walled (or smaller diameter) and multi-walled BNNTs [105]. Nevertheless, in the past 2–3 years, we have observed that the BNNT biomedical research community is shifting, using smaller diameter and higher quality BNNTs produced by BOCVD [115], laser evaporation PVC/HTP [116] or soon inductive thermal plasma methods. As of today the number of publications based on high-quality BNNTs is still limited, but the effect of nanotube quality and purity should be addressed in the near future. The emphasis on quality and purity factors will lead to more consistent or even more biocompatible biomedical applications.

## 6. Conclusions

In summary, recent advances in controlled synthesis of BNNTs by CCVD and mass production of BNNTs by the plasma-based techniques have started to stimulate significant progress on the investigation and application of BNNTs. These studies have led to further understanding of the growth mechanism of BNNTs and the large-scale production of high quality BNNTs. Together with BOCVD, increased availability of BNNTs opens up a wide-range of potential applications based on advantageous properties of BNNTs, including but not limited to composite materials, biomedical applications, novel nanoelectronic devices and aerospace technologies. Purification and functionalization are the critical factors and important for almost every application. Nevertheless, the advancement in BNNT synthesis and functionalization have started to stimulate and attract more interest in BNNTs. The future outlook of BNNT research is promising and exciting. Some of the important steps for further progress of BNNT research are including: (1) the exploration of new promising applications, and (2) the exploration of new synthesis technique for large-scale synthesis of high-quality and high-purity BNNTs.
